# Posttraumatic Stress Disorder: A Theoretical Model of the Hyperarousal Subtype

**DOI:** 10.3389/fpsyt.2014.00037

**Published:** 2014-04-04

**Authors:** Charles Stewart E. Weston

**Affiliations:** ^1^Independent Researcher, Marlborough, UK

**Keywords:** PTSD, fear, amygdala, insula, ACC, RDoC

## Abstract

Posttraumatic stress disorder (PTSD) is a frequent and distressing mental disorder, about which much remains to be learned. It is a heterogeneous disorder; the hyperarousal subtype (about 70% of occurrences and simply termed PTSD in this paper) is the topic of this article, but the dissociative subtype (about 30% of occurrences and likely involving quite different brain mechanisms) is outside its scope. A theoretical model is presented that integrates neuroscience data on diverse brain regions known to be involved in PTSD, and extensive psychiatric findings on the disorder. Specifically, the amygdala is a multifunctional brain region that is crucial to PTSD, and processes peritraumatic hyperarousal on grounded cognition principles to produce hyperarousal symptoms. Amygdala activity also modulates hippocampal function, which is supported by a large body of evidence, and likewise amygdala activity modulates several brainstem regions, visual cortex, rostral anterior cingulate cortex (rACC), and medial orbitofrontal cortex (mOFC), to produce diverse startle, visual, memory, numbing, anger, and recklessness symptoms. Additional brain regions process other aspects of peritraumatic responses to produce further symptoms. These contentions are supported by neuroimaging, neuropsychological, neuroanatomical, physiological, cognitive, and behavioral evidence. Collectively, the model offers an account of how responses at the time of trauma are transformed into an extensive array of the 20 PTSD symptoms that are specified in the *Diagnostic and Statistical Manual of Mental Disorders*, Fifth edition. It elucidates the neural mechanisms of a specific form of psychopathology, and accords with the Research Domain Criteria framework.

## Introduction

Posttraumatic stress disorder (PTSD) is a frequent mental disorder, with a lifetime prevalence in the US general population estimated at 6.8–7.8% (1, 2). In regions of civil disorder or armed conflict, rates are substantially higher (3, 4). PTSD is disabling, distressing, and commonly persistent (2, 5), and leads to serious impairment of economic and social functioning (6–9), as well as increased mortality from multiple causes (10). Although some interventions can be very effective, a substantial proportion of PTSD sufferers achieve limited improvement (11–13), so new and theory-based treatment options require early development. More generally, theoretical progress on this disorder may illuminate the mechanisms of anxiety disorders, the most prevalent class of psychological disorders, with a lifetime prevalence of 28.8% in the US general population (1).

The character of PTSD is well illustrated by the following vignette that epitomizes the condition of severely traumatized Vietnam veterans decades after active combat in Vietnam:

I can’t get the memories out of my mind! The images come flooding back in vivid detail, triggered by the most inconsequential things, like a door slamming or the smell of stir-fried pork. Last night, I went to bed, was having a good sleep for a change. Then in the early morning a storm front passed through and there was a bolt of crackling thunder. I awoke instantly, frozen in fear. I am right back in Vietnam, in the middle of the monsoon season at my guard post. I am sure I’ll get hit in the next volley and convinced I will die. My hands are freezing, yet sweat pours from my entire body. I feel each hair on the back of my neck standing on end. I can’t catch my breath and my heart is pounding. I smell a damp sulphur smell. Suddenly I see what’s left of my buddy Troy, his head on a bamboo platter, sent back to our camp by the Viet Cong. Propaganda messages are stuffed between his clenched teeth. The next bolt of lightning and clap of thunder makes me jump so much that I fall to the floor….” [(14), p. 470].

The Diagnostic and Statistical Manual of Mental Disorders, fifth edition (DSM-5) (15) and its predecessors have set out criteria for PTSD for over 30 years. Extensive research has generated a valuable body of findings, but current hypotheses of PTSD have yet to achieve a comprehensive account of the accumulated findings. Recent reviews [cf. Ref. (16–18)] explain the mechanisms of rather few of the 20 DSM-5 PTSD symptoms. Moreover, fear conditioning mediated by the amygdala dominates current PTSD theorizing, and is central to major theoretical models (19–22). Nevertheless, fear is not essential to the pathogenesis of PTSD, because there is good evidence that PTSD can develop in the absence of fear at the time of trauma [i.e., peritraumatic fear; Ref. (23–27)]. Together, it is suggested that a broader view of peritraumatic responses, and different theoretical perspectives, should enrich and progress understanding of PTSD.

A theoretical model is presented that elaborates how peritraumatic responses are transformed into an extensive array of DSM-5 PTSD symptoms, to manifest as the hyperarousal subtype of PTSD. In brief, the model elucidates how peritraumatic responses, particularly those participating in hyperarousal, are processed by the amygdala, which subsequently replicates those responses on later encounters with trauma-related stimuli, to produce hyperarousal symptoms. This perspective is consistent with grounded cognition, which contends that brain regions that are active during an event, are reactivated when that event is recalled, and is known to operate in diverse brain regions (28–32). Hyperarousal further drives the other symptom clusters (33, 34), and correspondingly, the amygdala modulates the activity of diverse brain regions known to be involved in PTSD. For example, there is an abundant literature that arousal-enhanced amygdala activity influences hippocampal function, and thus episodic memory performance [see for reviews, Ref. (35, 36) and summary later]. Similar amygdala processes are suggested to modulate diverse other brain regions involved in PTSD, namely, brainstem, visual cortex, rostral anterior cingulate cortex (rACC), and medial orbitofrontal cortex (mOFC). The result is a theoretical model, summarized in Figure 2, which integrates neuroscientific data on multiple brain regions, and extensive psychiatric findings on PTSD, to elucidate the etiology, symptomatology, and neurocircuitry of the hyperarousal subtype of PTSD, and to suggest new directions for theoretical, prevention, and intervention research.

## Major Features of PTSD

Posttraumatic stress disorder comprises four symptom clusters according to DSM-5, namely, hyperarousal, persistent re-experiencing of the trauma, avoidance of trauma-related stimuli, and negative alterations in cognitions and mood. Empirical findings have further characterized PTSD symptomatology, as summarized next. PTSD is a heterogeneous disorder, and there are several subtypes of it. One is characterized by hyperarousal, and a second by dissociation, numbness, and physiological unresponsiveness, accounting for approximately 70 and 30% of occurrences, respectively (37–39). Quite different brain mechanisms are likely involved (37–39), and only those relating to the former, hyperarousal subtype are considered, which is simply termed PTSD in this article. That is, the theoretical model elucidates the neural mechanisms of a specific form of psychopathology, and accords with the Research Domain Criteria (RDoC) framework (40, 41). Such PTSD represents the extreme of a continuum of stress reactions rather than a discrete syndrome (42–44). The etiology of PTSD is multifactorial (45, 46), and hyperarousal is of particular importance as detailed later. In contrast, peritraumatic intense fear commonly contributes to symptomatology, but is not a crucial factor as it is not experienced by a substantial minority of those who subsequently develop PTSD (23–25, 27). Pain, injury, and dysphoric emotions besides fear (e.g., shame, guilt, sadness, and anger) are further factors that can substantially contribute to PTSD development (24, 25, 47–50).

Peritraumatic hyperarousal is supported by the majority of behavioral and physiological findings (51–56). Reactivation of hyperarousal and other symptoms is elicited by specific trauma-related stimuli or reminders of them, even in conditions of no current danger (7, 18, 21, 57–59). Nevertheless, no significant differences between PTSD patients and controls are generally reported for physiological responses to control scripts, and to standard physical and mental challenges (58–61). Resting levels of physiological variables did not differ significantly between PTSD patients and controls in several studies that measured these variables under appropriate conditions [such as at home; Ref. (60, 62)]. More generally, the meta-analysis of Pole (63) found small and significant weighted mean effect sizes for resting heart rate (*r* = 0.18) and skin conductance level (*r* = 0.08) in PTSD, but not for other individual resting physiological variables. Basal levels of cortisol do not differ significantly between PTSD patients and controls, according to the meta-analysis of Meewisse et al. (64). There is, however, significant heterogeneity, and specific subgroups differ significantly from controls (64). More broadly, hyperarousal is the predominant PTSD symptom cluster (33, 34). A longitudinal study collected data on patients’ symptom levels through face to face interviews at several days, 3 and 12 months post-trauma. The findings were that hyperarousal strongly influences the development of the other symptom clusters but is little influenced by them (34). A subsequent study of similar design but with methodological modifications to increase generalizability, replicated the earlier findings (33).

Major sequelae of PTSD are disrupted economic and social behaviors, manifested in high levels of marital and parenting problems, divorce, unemployment, isolation, homelessness, and imprisonment (6–9). Comorbid disorders occur at high rates in PTSD sufferers (2, 65, 66). The relationship between PTSD and comorbid disorders appears complex and bidirectional. Specifically, a history of pre-trauma psychiatric disorders is a weak risk factor for PTSD (45, 46). Conversely, the time course of onsets suggests PTSD may often cause comorbid depression, anxiety disorders, and alcohol and other substance abuse/dependence disorders (65, 66). In sum, PTSD comprises a complex symptomatology, which continues to be elaborated and revised, and which must be explained by models of PTSD.

## PTSD Theoretical Model

**Figure 1:**
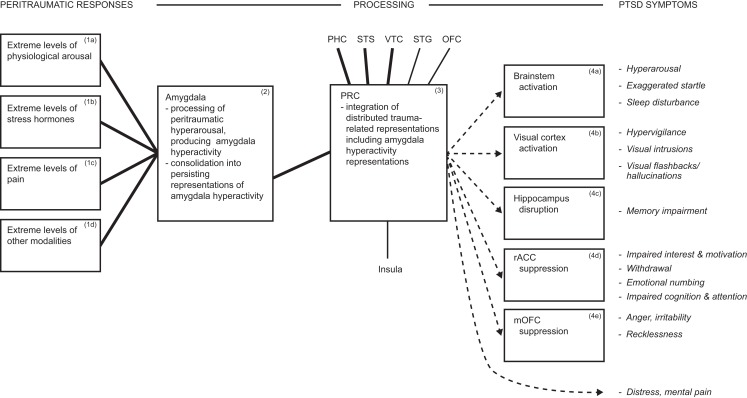
**Diagram summarizing hyperarousal processing by the amygdala, and consequent array of PTSD symptoms**. During traumatic interactions, the amygdala processes diverse hyperarousal inputs, producing amygdala hyperactivity. Consolidation in the amygdala generates persisting representations of hyperactivity, which participate in distributed trauma-related representations. Reminders reactivate the distributed trauma-related representations, including those mediated by the amygdala, which influence further brain regions and thereby drive multiple PTSD symptoms. E.g., amygdala projections to brainstem reactivate hyperarousal symptoms automatically and involuntarily. ▬, Heavy interconnections; ─, moderate interconnections; →, amygdala hyperactivity representations drive PTSD symptoms. Italics indicate symptoms. Abbreviations: dACC, dorsal anterior cingulate cortex; rACC, rostral anterior cingulate cortex; mOFC, medial orbitofrontal cortex; PHC, parahippocampal cortex; PRC, perirhinal cortex; STG, superior temporal gyrus; STS, superior temporal sulcus; VTC, ventral temporal cortex.

The model proposes that arousal/hyperarousal is processed by the (multifunctional) amygdala, which does so on grounded cognition principles, and these are summarized below. Hyperarousal is rarely defined, but simply means a high-level of physiological arousal. Arousal is a common component of diverse emotions, and may also occur in their absence (67–69). Dysfunctional hyperarousal is suggested to be the overriding feature of PTSD, and its independence of particular emotions is consistent with the diversity of peritraumatic emotions reported earlier. It is dysfunctional because its co-occurrence with traumatic reminders is automatic, involuntary, severe, and uncontrollable, even when there is no current danger or other challenge, as illustrated by the vignette quoted earlier, and as elucidated later. Besides processing hyperarousal symptoms, the amygdala also modulates the activity of multiple brain regions, thereby generating further DSM-5 PTSD symptoms. The hypothesis of the amygdala’s contribution to PTSD is summarized in Figure 1.

## Grounded Cognition

Grounded cognition operates widely in the brain (as do many other processes), including it is suggested, in the amygdala. It offers an account of the transformation of peritraumatic hyperarousal into enduring PTSD hyperarousal symptoms, and is the overall process that is performed in Box 2 of Figure 1. Grounded cognition proposes that enduring knowledge representations are grounded in or based in sensory, motor, and other specialized brain systems. Sensory systems, for example, which process the perception of a feature, also mediate enduring representations of that feature, usually in higher-level subregions. That is, sensory systems are engaged in both perceptual processing and in the mediation of sensory knowledge representations. Hence, a sensory system that is active during the perception of a stimulus, is later reactivated when the corresponding memory representation is retrieved. Similar principles apply to the motor system, which is involved in both action execution processes and in the mediation of action knowledge representations. In short, enduring knowledge representations are grounded in or based in the systems that mediate perceptual, motor, and other processes, hence the term grounded cognition (28–31). More broadly, a complex knowledge representation is constituted by a network of component representations distributed across multiple sensory, motor, and other specialized brain regions (28–31, 70, 71), and this is also termed a distributed representation. Further, such distributed networks may be integrated by means of nodes or hubs, which also efficiently reactivate such distributed networks so as to achieve knowledge reactivation and retrieval (28–31, 72).

Sensory property knowledge (e.g., object color knowledge) is represented in corresponding sensory systems (color perception system), and this is evidenced by diverse neuroimaging findings. In a neuroimaging study using functional magnetic resonance imaging (fMRI), healthy subjects were scanned while they performed a color perception task (Farnsworth–Munsell 100 hue test) and a color knowledge task (a property verification task). The findings were that partially overlapping subregions of the color processing system mediated color perception and color knowledge representation (32). Findings that likewise support overlapping of perceptual processing and of knowledge representation within a sensory modality, have been reported by a number of neuroimaging studies using various experimental designs, and covering multiple sensory modalities including visual object form, auditory, somatosensory, and gustatory modalities (73–75).

Action knowledge is likewise represented in the motor system and participates in distributed knowledge representations. This is exemplified by the distributed networks that represent tool concepts. The robust findings of numerous neuroimaging studies using diverse designs are that tool concepts are represented across multiple brain regions, including ventral premotor cortex, and intraparietal sulcus in parietal cortex. These regions likely represent the typical actions performed with tools, and contribute to tool identification (76). Convergent support is provided by neuropsychological findings that motor system dysfunction is associated with impaired action knowledge. Several studies of substantial groups of patients (*n* = 29 and *n* = 90) with circumscribed brain lesions, presented diverse tests of action knowledge, and mapped lesion locations from structural MRI data. The findings were that impaired knowledge of actions was associated with lesions to motor cortical areas, and regions interconnected with them (77, 78). In summary, numerous brain systems that mediate perceptual or motor processes also mediate corresponding persisting knowledge representations, in accordance with grounded cognition. It is suggested that the amygdala implements corresponding operations, as described in the next sections.

## The Amygdala, Peritraumatic Hyperarousal, and Hyperarousal Symptoms

The amygdala and its projections are crucially involved in PTSD symptomatology, and this is evidenced by a neuropsychological study. The study used Vietnam Head Injury Study (VHIS) participants who had experienced heavy combat exposure, and had been assessed for PTSD using standard methods. The findings were that of the combatants with amygdala lesions (*n* = 15), none developed PTSD, whereas in comparison groups of combatants with lesions to control regions or no brain lesions, 40–48% developed PTSD (79).

The model contends that the amygdala, which processes arousal (80, 81), also produces related representations on grounded cognition principles. Correspondingly, at the severe intensity levels that characterize PTSD, peritraumatic hyperarousal is transformed into enduring hyperarousal symptoms. In more detail, multiple forms of arousal and peritraumatic responses (Boxes 1a–d, Figure 1) input to the amygdala, enhancing amygdala activity (Box 2, Figure 1). Consolidation is implemented in the amygdala to produce enduring representations of amygdala hyperactivity (Box 2, Figure 1). Amygdala-mediated representations participate in and are reactivated with distributed stimulus representations (Box 3, Figure 1), and via projections to brainstem can reactivate arousal/hyperarousal (Box 4a and related symptom, Figure 1). The result in PTSD is hyperarousal that is automatic, involuntary, and severe, even in conditions of no current danger or other challenge. Arousal that is integral with challenging stimulus representations mobilizes necessary resources, so is normally adaptive [cf. Ref. (81)], but PTSD is the extreme of the stress reactions continuum (42–44), so the effects are extreme and disabling, and constitute hyperarousal symptoms.

### Arousal/hyperarousal processing in the amygdala

The amygdala receives extensive arousal/hyperarousal inputs (Boxes 1a–d, Figure 1), which is evidenced by neuroanatomical, neuroimaging, and physiological findings. The cardiovascular and respiratory systems project via multiple pathways to the amygdala in rodent and monkey. Visceral sensory nerves of the autonomic nervous system (ANS) that innervate these organs project to spinal cord neurons, which relay information sequentially via thalamus and insula to the amygdala. A major medullary pathway has similar origins, and projects sequentially through the visceral region of the nucleus of the solitary tract (NTS) in the medulla, parabrachial complex (PBC), thalamus, and insula to multiple amygdaloid nuclei (82–85). Functional activation of the human respiratory system can be elicited by such methods as adding resistive respiratory loads to an external breathing circuit, and the resulting brain activations have been neuroimaged. A common finding of such studies is that the activated brain regions include the amygdala (86–89).

The hypothalamus is a further arousal-related system, which projects substantially to multiple amygdaloid nuclei, in rodent neuroanatomical studies (84). Consistent with the neuroanatomy, electrical stimulation of parts of the hypothalamus and PBC was found to evoke responses in amygdala neurons, predominantly in the central nucleus (90).

Elevated levels of stress hormones also influence amygdala activity. Cortisol binds directly to glucocorticoid receptors on the amygdala, and to glucocorticoid receptors on NTS neurons, which project to the amygdala as described above (35, 85, 91). Epinephrine binds to vagus neurons of the parasympathetic division of the ANS, which projects to the amygdala via routes described above (35, 85, 91–93). Consistent with the neuroanatomy, electrical stimulation of vagus neurons in rats was found to produce substantially enhanced neurotransmitter release in the basolateral amygdala (94).

In human neuroimaging studies, enhanced levels of arousal are brought about by stimuli such as pictures or words, and these have been found to elicit enhanced amygdala activation (95–98). In PTSD, the occurrence of peritraumatic hyperarousal, often assessed hours or days after trauma, is commonly supported by studies of heart rate, respiration rate, and subjective arousal (51–56). Moreover, elevated heart rate and respiration rate shortly after the trauma significantly predict subsequent PTSD diagnosis or PTSD symptoms (52, 53, 55, 56). In addition, a linear relationship between severity of combat as measured by the number of firefights and prevalence of PTSD diagnosis, was found in military personnel deployed to Iraq (99). This finding was replicated in personnel deployed to Afghanistan (99). Further, elevated pre-trauma amygdala reactivity predicts a greater increase in PTSD symptoms after exposure to stressful events (100). Peritraumatic hyperarousal is likely relayed to the amygdala by the pathways described above; nevertheless, empirical verification of this in human is required.

### Consolidation in the amygdala

Consolidation is the process whereby initially transient neural activity is transformed into persisting neural representations (101–103), and there is substantial evidence it occurs in the amygdala (Box 2, Figure 1). At the neural level, consolidation occurs in the period after learning events (101–103), and corresponding processing occurs in the amygdala. In a lever-pressing paradigm, cats that had already learned to lever-press received on a single trial a footshock. Neurons in several amygdaloid nuclei were recorded and the findings were that their activity increased in the period after the footshock for several hours (mean 137 ± 5 min) relative to control levels (104).

Long-term potentiation (LTP) is a persistent enhancement in a neuron’s responsiveness (105), and is thought to be a major mechanism that contributes to the formation of long-term representations and memories (106). There is substantial evidence for its occurrence in the amygdala. For example, rats were trained with a classical fear conditioning design, involving a tone repeatedly paired with electric shock, and neural activity was measured in the amygdala. The findings were that learning that the tone predicted shock was paralleled by persisting enhancements in responses of amygdala neurons to the tone (107). Moreover, such enhanced responsiveness corresponds to that brought about in the same amygdala areas through the experimental induction of LTP (108). Results consistent with these mechanisms and related ones have been reported by multiple studies (109–112). In addition, fear-related conditioning in rats can be severely impaired by infusing into the amygdala pharmacological agents that block LTP and other processes in the consolidation cascade (110, 113–115). Moreover, destruction of parts of the amygdala well after fear conditioning training has been completed, abolishes that fear-related conditioning (116–118). Collectively, the hypothesis that the amygdala implements consolidation is well supported.

### Amygdala-mediated representations participate in distributed representations

Amygdala-mediated representations participate in distributed representations, and via projections to brainstem can reactivate arousal/hyperarousal (Boxes 3, 4a, and related symptom, Figure 1). The multiple components of distributed representations are linked together by perirhinal cortex [PRC; Brodmann’s Areas 35, 36, and part of 38, which constitutes the temporal pole; Ref. (119, 120)]. The binding role of PRC is evidenced by robust findings that lesions to PRC produce visual recognition and visual discrimination impairments for complex visual stimuli, but not for more basic ones, in monkey and human (72, 121–124). PRC interconnects heavily with ventral temporal cortex (VTC), superior temporal sulcus (STS), parahippocampal cortex, and amygdala, and moderately with superior temporal gyrus (STG), insula, and OFC, according to neuroanatomical tracing studies in monkey (120, 125). Thus, amygdala–PRC interconnections, which are dense, pervasive, and reciprocal in primate and rat (83, 84, 125), enable the amygdala to contribute specialized representations to distributed representations in parallel with other regions. Moreover, neuroimaging investigations of the neural representation of diverse objects, reveal that such neural representations are constituted by the above regions, including the amygdala [see for reviews, Ref. (30, 71)].

In PTSD, a common neuroimaging paradigm involves presentation of individualized auditory scripts of highly traumatic and of neutral experiences to subjects while they are neuroimaged. Common findings are that relative to comparison groups or conditions, PTSD patients during traumatic recollections, which are likely constituted by distributed trauma-related representations [cf. Ref. (63)], exhibit enhanced activations in the amygdala, as well as other brain regions [see for reviews and meta-analyses, Ref. (16–18, 21, 126)]. Further, amygdala involvement in distributed representations suggests that such amygdala activation should be automatic, and this is supported by findings from neuroimaging studies of amygdala activation during unattended processing of feared stimuli (127, 128). Thus, convergent evidence supports amygdala participation in distributed representations, including trauma-related ones.

The amygdala can reactivate arousal/hyperarousal, via its heavy projections to hypothalamus and brainstem. Specifically, the amygdala sends heavy projections to hypothalamus, NTS, and PBC, and it projects extensively to further brainstem regions (14, 84, 92, 129, 130). Convergent support is provided by findings from neuroimaging studies that PTSD symptom severity is positively correlated with amygdala activity, as measured by PET or fMRI during exposure to trauma-related or experimental stimuli (131–135). Additional brain regions, specifically insula (BA 13), anterior parts of ACC (BAs 25, 32), and dorsomedial PFC (BAs 9, 10) may also contribute to components of hyperarousal (136, 137).

Several cautions concerning the above hypotheses and evidence should be noted. Enhanced peritraumatic stress hormone levels are not supported by recent studies (138, 139); nevertheless, field studies of stressed animals demonstrate enormous increases (140–142). The contradictory findings may arise because of diverse methodological issues. For example, human stress hormone measurements were typically taken several hours after trauma, whereas stress hormone half-lives measured in rat blood range from 70 s to 20 min (143–145). Further, stress hormone levels are strongly influenced by psychological factors [e.g., Ref. (146, 147)], so are likely to have been lowered by such factors as being rescued, knowing that medical care and other support is at hand, and so forth. As regards measures such as resting heart rate, these are typically taken hours or days after trauma, so are only coarse approximations of peritraumatic processes, and are unlikely to reflect the intensity of responses involving, for example, faintness, vomiting, and defecation, as can occur in combat (148). Medication administered during ambulance transport or in the emergency room are further issues.

There is considerable variability in neuroimaging findings. Nevertheless, such variability has been ascribed to numerous technical reasons, including low spatial and temporal resolution of scanners, limited subject numbers, lack of segregation of the several subtypes of PTSD, comorbid disorders, the amygdala’s deep location, small size and signal changes, and so forth [see for reviews, Ref. (18, 126)]. Brainstem regions probably participate in the reactivation of hyperarousal co-occurring with trauma-related stimuli, but brainstem activations are rarely reported in neuroimaging studies of PTSD, perhaps again for technical reasons. Amygdala hyperactivity could be a pre-trauma vulnerability factor which with others could substantially explain PTSD symptomatology (100, 149). Nevertheless, several meta-analyses, twin, and other studies have found that pre-trauma vulnerability factors have generally small effects, which interact with the crucial traumatic events in the generation of PTSD symptomatology and amygdala hyperactivity (45, 46, 49, 150–152).

In summary, hyperarousal is the predominant symptom of PTSD (33, 34), and it is suggested to be processed by the amygdala. Specifically, peritraumatic hyperarousal is transformed into enduring hyperarousal symptoms, and this is likely mediated by a network comprising the amygdala, its inputs, and its interconnections with PRC and brainstem.

## The Amygdala Modulates Diverse Brain Regions, so Driving Further PTSD Symptoms

The primate amygdala interconnects with approximately 90% of cerebral cortical areas (153), and numerous subcortical areas (92, 130). It is suggested amygdala hyperactivity modulates the activity of diverse interconnected brain regions, and thereby drives the development of further DSM-5 PTSD symptoms, which are generally markedly different in nature from hyperarousal. This is summarized in Boxes 4a–e and related symptoms, Figure 1.

### Exaggerated startle, sleep disturbance, and modulation of further brainstem regions

There is abundant evidence that the amygdala crucially participates in the enhancement of startle [see for reviews, Ref. (14, 154)], so amygdala hyperactivity likely produces the exaggerated startle symptom of PTSD (Box 4a and related symptom, Figure 1). Startle is elicited by a sudden unexpected stimulus, such as a noise, and involves involuntary reactions by muscular, hormonal, and visceral systems (105). In laboratory paradigms, heightened startle responses are reliably elicited by presentation of conditioned stimuli (e.g., a light formerly paired with electric shocks) and an unexpected sudden stimulus (e.g., a noise burst); together these constitute the fear-potentiated startle paradigm (14, 154). The network underlying such heightened startle responses involves a major modulatory role for the amygdala and its brainstem projections (14, 154). This claim is evidenced by findings that in rodents trained and tested in the fear-potentiated startle paradigm, lesions of the amygdala consistently abolish the heightened fear-potentiated startle response (14, 154). Conversely, low-level electrical stimulation applied to the rodent amygdala heightens the startle response, but when applied to adjacent brain regions, does not do so (14). Moreover, such amygdala stimulation enhances activation of the brainstem region that implements the startle response (14, 154). Thus, the amygdala and its brainstem projections can enhance startle responses. It is suggested that amygdala hyperactivity in PTSD and the consequent brainstem activation, likely produce the exaggerated startle symptom of PTSD.

The amygdala likely participates also in the sleep disturbance symptom of PTSD [(155); Box 4a and related symptom, Figure 1]. The networks that regulate wakefulness and sleep substantially overlap ANS regions that modulate arousal (156). These shared regions include midbrain reticular formation, NTS, posterior hypothalamus, anterior hypothalamus, and preoptic area (156). The amygdala interconnects with components of the wakefulness and sleep regulating networks, including brainstem subregions, NTS, anterior hypothalamus, preoptic area, basal nucleus of Meynert, and OFC (84, 130, 155–157). Non-rapid eye movement (NREM) sleep is a state of attenuated arousal, during which overall brain activity is reduced substantially, according to neuroimaging findings in healthy human subjects (155, 158, 159). After controlling for this overall decline, brainstem subregions that generate and maintain sleep, as well as the amygdala and other areas, are relatively activated compared to waking (155, 156, 159). NREM sleep recurs throughout the sleep period, in alternation with rapid eye movement (REM) sleep. The latter is a state of heightened activity in numerous brain regions and deactivations in others. There is increased activity relative to wakefulness and NREM sleep, in brainstem subregions, amygdala, and other regions, whereas high-level sensory and lateral prefrontal cortices are deactivated (155, 157, 158, 160).

It has been hypothesized that amygdala hyperactivity in PTSD likely drives the sleep disturbance symptom of this disorder, through modulation of components of the wakefulness and sleep networks (155). Specifically, amygdala hyperactivity likely activates brainstem subregions promoting wakefulness, and reduces activation of brainstem subregions that generate and maintain sleep (155). The amygdala also suppresses mOFC activity as evidenced in a later section. OFC importantly influences sleep; electrical stimulation of it in animals produces sleep, and neuroimaging studies of humans report increased activation during NREM and REM sleep. Furthermore, lesions of OFC in experimental animals reduce sleep (156, 157). Thus, amygdala suppression of mOFC likely further disturbs sleep. The amygdala may also modulate other components of the wakefulness and sleep networks, although further investigations are required. In sum, amygdala hyperactivity modulates brainstem subregions, mOFC, and perhaps other components of the networks that regulate wakefulness and sleep, and so may drive the sleep disturbance symptom of PTSD.

### Hypervigilance, visual intrusions, and flashbacks and modulation of visual cortex

Posttraumatic stress disorder symptoms that are primarily visual symptoms, including visual sensory components of flashbacks, are likely mediated by a network including visual cortex, a region which is modulated by the amygdala (Box 4b and related symptoms, Figure 1). Visual perceptual processing in PTSD is enhanced for trauma-related stimuli (161). This is evidenced by findings that identification accuracy is higher for trauma-related stimuli than control stimuli, in PTSD patients but not healthy trauma-exposed comparison subjects (161). Moreover, the authors suggest that such enhancement likely contributes to intrusions and other re-experiencing PTSD symptoms. Several neuroimaging studies have examined brain activations during traumatic flashbacks or analogs of them. The findings were that visual flashbacks and their analogs consistently recruited early visual cortex; less consistent activations were reported in somatosensory cortex, insula, and motor-related areas (162–164). A further neuroimaging study using fMRI examined the non-traumatic visual hallucinations that are the hallmark of Charles Bonnet syndrome patients, who are otherwise neuropsychiatrically normal, and the related brain activations. The findings were that visual hallucinations of particular stimuli (e.g., color, faces) co-occurred with activations of visual cortical subregions that are specialized for representing those stimuli (165). Convergent evidence for visual cortex involvement in PTSD is provided by a longitudinal study of brain structure changes in veterans with and without PTSD. The findings were that significantly greater atrophy of visual cortex (and other brain regions) occurred in veterans with PTSD whose symptoms were worsening, whereas such atrophy did not co-occur in those whose PTSD symptoms were improving. These findings may arise from the neurotoxic effects of overactivation (166). Thus, the findings of the limited investigations to date suggest that enhanced activation in visual cortex occurs in PTSD, and likely contributes to the visual sensory symptoms of PTSD.

The amygdala likely drives enhancements of visual cortex functioning. The amygdala projects densely to all levels of the ventral visual stream, from anterior TE to posterior V1, according to neuroanatomical tracing studies in monkey (167–169). Moreover, examination of amygdaloid boutons adjacent to TE and V1 neurons, revealed that the boutons formed asymmetric synapses and predominantly onto dendritic spines. Such synapses are normally excitatory, suggesting amygdala-visual cortex projections enhance visual function (170). This is further supported by behavioral and neuroimaging studies. In an attentional blink paradigm, neutral and negative words were briefly presented at short temporal intervals, and the findings were that healthy controls processed negative words with greater accuracy than neutral words. This effect, however, was not found in patients with bilateral or left amygdala lesions (171). In an fMRI study, fearful faces relative to neutral faces elicited enhanced activation in visual cortical areas in healthy controls. Such visual cortex enhancement, however, did not occur in patients who had amygdala lesions due to sclerosis, but structurally intact visual cortex (172). Together, these findings are consistent with amygdala dependent enhancement of visual function.

Amygdala modulation of visual cortex may also have more fundamental plasticity effects. In a rat study, a training pure tone was presented on 30 trials in a single session, concurrently with electrical stimulation of the basolateral amygdala. A microelectrode array implanted in the primary auditory cortex (A1) measured plasticity changes in that system. The findings were that relative to pretraining baseline measurements, the frequency tuning of a majority of the tested A1 sites shifted toward or to match the frequency of the training tone. These changes were specific to the training tone, as they failed to occur for a tone that was not accompanied by basolateral amygdala stimulation. Such plasticity developed rapidly after a single training session, underwent consolidation processes, and was enduring as it persisted throughout the 3-week duration of testing. There was also some evidence for increased sensitivity (reduced threshold) and increased selectivity (reduced bandwidth) at these A1 sites, although these effects were less enduring (173, 174). Plasticity is ubiquitous in the brain, so corresponding plasticity likely occurs in human visual cortex.

Collectively, multiple findings suggest that trauma-specific hyperactivity of the amygdala in PTSD likely drives enhancement of visual perceptual processing, as well as specialization and enlargement of visual cortex representations, of trauma-related stimuli. These effects likely support hypervigilance, visual intrusions, and visual flashbacks/hallucinations symptoms of PTSD, although further investigations in human are required. In addition, the amygdala projects to further unimodal sensory regions (130, 167, 168), and likely replicates parallel effects in those sensory domains also.

### Memory impairment and modulation of the hippocampus

The hippocampus is disturbed in PTSD, likely producing memory impairment for important aspects of the traumatic event, a DSM-5 PTSD symptom [(17, 18, 21, 126); Box 4c and related symptom, Figure 1]. Numerous functional neuroimaging studies have reported aberrant activations of the hippocampus in PTSD, with reports of both diminished and increased activations, although the reasons for these variations are not clear (17, 18, 21, 126). In addition, structural neuroimaging studies commonly report reduced hippocampal volume (18, 21). In a VHIS study, a patient group was identified with damage to the hippocampus but none to the amygdala (*n* = 9). Four of these patients had developed PTSD, a prevalence that was not significantly different from that of the control groups (79). These findings suggest that the hippocampus does not play a crucial pathogenic role in PTSD; it is further suggested that hippocampal dysfunction is probably driven by amygdala influence, as elucidated below.

The hippocampus is a high-level region in a hierarchy of interconnected brain regions, which together support progressively more comprehensive representations, which are used in perception, memory, cognition, and so forth. The particular function of the hippocampus is to integrate complex representations from earlier regions (e.g., PRC and entorhinal cortex) with spatio-temporal representations, to generate episodic memory [i.e., event memory; Ref. (72, 175, 176)]. Thus, impaired memory for important aspects of the traumatic event is likely brought about by hippocampal dysfunction.

Numerous studies have provided evidence that the amygdala influences the hippocampus. The primate amygdala sends heavy anatomical projections directly and extensively to the hippocampus, and indirectly via the entorhinal cortex, a major input to the hippocampus. The reciprocal projections are substantially weaker and restricted in extent (83, 92, 130, 168). Functionally, the amygdala enhances hippocampal consolidation of emotionally arousing information, leading to greater memory for such information. This is supported by abundant evidence from neuroimaging, neuropsychological, behavioral, and animal studies (35, 36, 177–179).

Nevertheless, the amygdala may also impair memory retrieval processes in the hippocampus, and these effects have many parallels with the memory enhancement effects; that is, participation of stress hormones, common neurotransmitters, and restriction to emotionally arousing memories (177, 179). A neuroimaging study found that the amygdala and hippocampus participate in the recollective retrieval of emotional relative to neutral memories. Further, correlation analyses revealed that the amygdala and subregions of hippocampus were strongly coactivated during such recollection of emotional memories (180). A number of studies have presented stressful challenges that elicit endogenous release of stress hormones or have directly administered glucocorticoids (e.g., cortisol), to healthy human subjects before memory testing. The findings were that memory retrieval was impaired during arousal and elevation of stress hormone levels, predominantly for emotionally arousing information (179, 181–184). Such retrieval deficits, however, may be modulated by multiple factors, such as duration and extent of hormone elevations, timing and type of retrieval tests, and so forth (177, 183). The amygdala is responsive to stress hormones as described earlier, and releases noradrenaline. Consistent with the involvement of this system, cortisol induced memory impairment is abolished by treatment with propranolol, an antagonist of adrenergic receptors (182). In addition, further neuroimaging studies found that elevated cortisol levels reduce activation of parts of the medial temporal network during retrieval of memories (185, 186).

Much of the evidence above, as well as the amygdala’s peritraumatic involvement, the amygdala’s established modulation under stress of hippocampal consolidation, and the latter phenomenon’s parallels with retrieval impairment, suggests the amygdala likely modulates hippocampal retrieval rather than the reverse. Further findings are that hippocampal volumes are commonly reduced in severe PTSD (18, 21, 187), and that reduced hippocampal volume is a risk factor for PTSD (187). The theoretical implications of these findings are currently unclear.

Collectively, there is abundant evidence that amygdala activity can enhance hippocampal consolidation, and these brain regions likely participate also in stress-induced impairment of emotional memory retrieval. The evidence generally suggests the amygdala may drive hippocampal dysfunction to produce the PTSD symptom of impaired memory for important aspects of the traumatic event, although further studies are needed.

### Impairments of motivation, emotion, and cognition, and modulation of rostral anterior cingulate cortex

The rostral (anterior) subregion of anterior cingulate cortex (rACC), and adjacent medial subregion of orbitofrontal cortex (mOFC) are often treated together as ventromedial prefrontal cortex (vmPFC). Nevertheless, as these regions have both commonalities and substantial differences in connectivity, functions, and contributions to PTSD symptomatology, they are reviewed separately. The rACC participates in PTSD, likely producing impairments of motivation, emotion, and cognition (Box 4d and related symptoms, Figure 1). Moreover, impaired motivation and related symptoms likely contribute to the social and economic sequelae of PTSD (6, 9), adding to their significance. The rACC manifests in PTSD diminished responsiveness to trauma-related stimuli, which is a robust finding of functional neuroimaging studies (16–18, 126). Such diminished activation is inversely correlated with PTSD symptom severity (134, 188–190). In addition, following therapy rACC activity increases and this is associated with reduction of PTSD symptoms (191, 192). Together, these findings indicate that rACC is hypoactive in PTSD, and that such hypoactivation likely contributes substantively to PTSD symptomatology.

The normal ACC participates in networks that process wide-ranging motivated behaviors, emotion, cognition, and attention (193–195). In addition, lesions, degeneration, or hypometabolism located predominantly in rACC have been associated with impairments of these functions, including motivational and emotional deficits such as apathy, disinterest, aimlessness, inactivity, and uncommunicativeness (196–200). PTSD patients manifest DSM-5 symptoms of impaired interest and motivation, withdrawal and detachment, and emotional numbing [collectively termed numbing symptoms; Ref. (26, 201)], as well as impaired cognitive and attentional functioning according to neuropsychological tests (202–204). There is evident overlap between impairments caused by rACC pathology, and multiple PTSD numbing symptoms, suggesting rACC dysfunction participates in the latter also. Convergent evidence is provided by an fMRI study of numbing symptoms in women with PTSD predominantly due to childhood abuse. The findings were that emotional numbing symptoms in the PTSD group relative to healthy controls were associated with hypoactivation of rACC and adjacent medial PFC (205).

The diminished rACC activation in PTSD is inversely correlated with amygdala activation according to the meta-analysis of Hayes et al. (16), suggesting one region may functionally influence the other. Contrary to the conventional view, it is suggested the predominant influence is that trauma-specific amygdala hyperactivity in PTSD likely drives the diminution of rACC activity, and consequent impairment of rACC functions. The ACC is reciprocally interconnected with the amygdala in primates; ACC (mainly BAs 24, 25) projections to the amygdala are moderate, whereas amygdala projections to ACC (BAs 24, 25, 32) are dense throughout the ventral to dorsal extent of rACC (130, 168, 206). In addition, in rodent experiments, amygdala activation elicited by fear conditioned stimuli or by electrical stimulation, suppresses rACC and medial PFC activity (207, 208). Further, in an auditory oddball task, targets that elicited arousal as measured by skin conductance responses, were associated with reduced activation in rACC as measured by fMRI, in PTSD subjects relative to controls. In contrast, non-arousing targets had no such rACC effects (209). Together, these findings support the amygdala’s suppressive influence on rACC. Consistent with the overall hypothesis of this section, Litz et al. (201) found that, after controlling for demographic variables, trauma exposure, comorbid disorders, and PTSD symptom clusters, hyperarousal symptoms were the best predictor of numbing symptoms. Moreover, these findings were supported in subsequent studies of diverse trauma samples by the same and other research groups [e.g., Ref. (210, 211)]. In sum, trauma-specific amygdala hyperactivity, which derives from peritraumatic responses, likely drives the diminished activity of rACC. This diminished activity likely contributes to the motivational, emotional, cognitive, and attentional symptoms of PTSD and their sequelae.

### Anger, irritability, aggression, and recklessness, and modulation of medial orbitofrontal cortex

Anger, irritability, and aggression are closely related, and anger/irritability may be expressed in multiple forms including as aggression, or in extremes as violence (212–214). In addition, restraints on angry impulses are an important component of anger (212–214). The mOFC is located adjacent to rACC on the vmPFC. In PTSD, mOFC is hypoactive, which is suggested to be driven by amygdala hyperactivity, and it crucially participates in anger, irritability, aggression, and recklessness symptoms of PTSD (Box 4e and related symptoms, Figure 1). There are many similarities in findings between mOFC and rACC regions. The mOFC manifests in PTSD reduced activation to trauma-related stimuli (16, 17, 126). The mOFC is substantially and reciprocally interconnected with the amygdala, according to neuroanatomical tracing studies in monkey (168, 215, 216). In addition, mOFC hypoactivation is inversely correlated with amygdala activation according to the meta-analysis of Hayes et al. (16). Further, electrical stimulation of the rat amygdala suppresses activity in medial PFC (208). Together, these similar findings suggest that the amygdala may suppress mOFC activation, paralleling the amygdala’s suppression of activation of the adjacent rACC.

Impaired function of mOFC produces behavioral disinhibition, which is the expression of socially inappropriate and aberrant behaviors, as well as impaired regulation, and these likely participate crucially in anger/aggression (217), and recklessness. In a study of patients with frontotemporal dementia, behavioral disinhibition, which was measured with the Neuropsychiatric Inventory, was found to be significantly correlated with mOFC hypometabolism, which was measured with PET and the [18F]fluorodeoxyglucose method (218). A further study of patients with dementia used the same behavioral measures, together with structural MRI and voxel-based morphometry. A significant association was found between reduced gray matter tissue in mOFC and several further regions, and several behavioral impairments including disinhibition (200). Lesions to the OFC have also been acquired through head injuries, and such patients have been found to manifest elevated levels of anger and aggression as well as aberrant social behaviors (219, 220). For example, in the latter study of a large sample of VHIS patients, elevated levels of aggression were found in patients with lesions to vmPFC, relative to patients with lesions to other brain regions and to healthy controls (220).

A different experimental paradigm compared patients with major depressive disorder with anger attacks, patients with major depressive disorder without anger attacks, and healthy controls. The study involved individualized anger and neutral scripts, an adaptation of the Pitman et al. (61) paradigm, and PET neuroimaging. The findings were that depressive patients with anger attacks relative to controls, during anger provocation manifested hypoactivation of mOFC and adjacent medial BA 10 (221). In an fMRI neuroimaging study, healthy subjects recalled anger eliciting events, and executed specified anger regulation strategies. The findings of the conjunction analysis were that anger regulation strategies activated mOFC and adjacent OFC regions, lateral PFC, and additional regions outside PFC (222).

Taken together, there is convergent evidence from diverse experimental paradigms that the OFC, particularly the mOFC, mediates inhibitory and regulatory processes (among other functions), and impairment of these leads to elevated levels of disinhibition, anger, and aggression. In PTSD, amygdala hyperactivity likely drives suppression of mOFC activity, which contributes to the corresponding symptoms of anger, irritability, and aggression. This overall hypothesis is further supported by behavioral findings that hyperarousal is robustly and significantly associated with measures of anger and aggression in PTSD (223, 224).

Recklessness or self-destructive behaviors are heterogeneous, and include diverse potentially destructive activities: sexual excesses, risky driving, substance abuse, aggression, violence, and so forth (225). Such diversity is likely promoted by multiple and poorly understood mechanisms. Recklessness or self-destructive behaviors are evidently related to aggression, which includes self-directed injurious or destructive activities (217). Similarly, impaired inhibitory and regulatory processes, as well as impaired processing of adverse feedback and of risk, are likely components for such behaviors to become manifest, and these flow from mOFC disruption [see earlier; Ref. (226, 227)]. Hence, mOFC dysfunction may play a crucial role in this PTSD symptom also. In sum, amygdala hyperactivity likely drives mOFC hypoactivation, which likely participates in anger/aggression, and recklessness symptoms of PTSD.

### Pain, distress, and the amygdala

Severe pain importantly contributes to PTSD development (47, 49, 50), and the amygdala may process this similarly to hyperarousal (Figure 1). The amygdala receives heavy nociceptive inputs via several neuroanatomical pathways according to neuroanatomical tracing studies in monkey and rodent. Nociceptors in skin and throughout the body project to spinal cord neurons, which relay nociceptive information via dense projections sequentially through spinal cord, several nuclei of the thalamus, insula, or to SII which in turn projects heavily to insula, thence to multiple amygdaloid nuclei (83, 84, 228, 229). A further pathway relaying nociceptive information involves similar origins that project via NTS, PBC, to the central amygdaloid nucleus (228, 229). Human neuroimaging studies further demonstrate that painful stimulation activates the amygdala, and multiple additional areas (230–233). Nevertheless, lesions to the amygdala do not affect pain sensitivity levels nor latency of pain responses (129, 234, 235), so the amygdala may mediate chronic or high-level pain-related functions. Pain-related amygdala activity undergoes consolidation (236–238), and may produce noxiousness representations that participate in distributed representations. Mental pain or distress and physical pain are processed by largely overlapping networks of brain regions (239–241), including the amygdala (241), so mental pain also may be processed similarly to hyperarousal. Pain processes are normally adaptive, but in PTSD are likely extreme and consequently disabling.

To summarize the above sections, the amygdala interconnects with and modulates the activity of multiple brain regions, and thereby drives the development of diverse DSM-5 PTSD symptoms. This is consistent with behavioral findings that hyperarousal drives the development of the other PTSD symptom clusters (33, 34).

## Fear and the Insula

**Figure 2:**
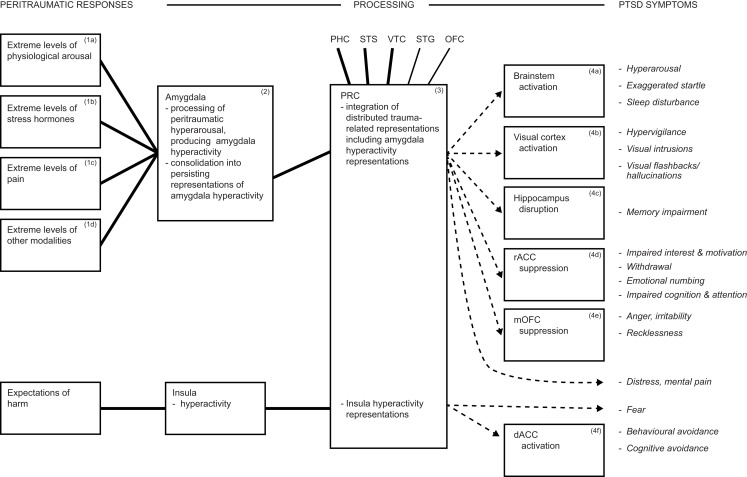
**In addition to amygdala processing, the insula participates in the processing of fear and likely drives avoidance symptoms**. Together, these mechanisms account for some 80% of DSM-5 PTSD symptoms. Abbreviations: dACC, dorsal anterior cingulate cortex; rACC, rostral anterior cingulate cortex; mOFC, medial orbitofrontal cortex; PHC, parahippocampal cortex; PRC, perirhinal cortex; STG, superior temporal gyrus; STS, superior temporal sulcus; VTC, ventral temporal cortex.

Persistent dysphoric emotions such as fear are a further DSM-5 PTSD symptom. Theorizing about fear has been dominated by the amygdala’s contribution [see for reviews, Ref. (111, 154)], but the amygdala solely cannot account for fear. The amygdala participates in fear, but also in diverse other emotions (81, 231, 232, 242, 243). In addition, fear is frequently elicited in laboratory animals by inflicting pain, and the latter is also processed in the amygdala (see earlier), suggesting the operation of possible confounds. Furthermore, rodents with amygdala lesions manifest impaired fear conditioning when assessed with fear-potentiated startle or freezing, yet largely intact fear conditioning when assessed by avoidance behaviors (35, 244, 245). Likewise, several neuropsychological studies have found that patients with bilateral or unilateral amygdala lesions continue to experience apparently normal subjective fear and anxiety, although with some subtle abnormalities of emotion generally (246, 247). Similarly, in a VHIS study using the Structured Clinical Interview for DSM-IV-TR disorders, it was found that the frequency of any fear and anxiety disorders (which did not include PTSD) was not significantly different among patient groups with lesions to the amygdala or to control regions (79). Thus, additional brain regions are crucial for the processing and representation of fear. In particular, the insula is a region that is reliably activated in PTSD and in anxiety disorders (18, 126), and it may contribute to the mediation of the intense and persistent fear symptom of PTSD (Figure 2).

Fear is poorly understood, but is widely regarded as elicited predominantly by danger, real, or perceived (105, 248–250). Nevertheless, this assumption is rarely tested and is challenged by empirical findings. Several studies of military personnel have demonstrated the co-occurrence of high danger levels, but little subjective fear or physiological activation (251, 252). In addition, correlations between dangerousness and fear were found to be low and non-significant in parachute trainees (253). Instead, expectations of harm may be the chief elicitors of fear (254, 255). Hence, dangers that can be controlled, normally do not cause harm nor elicit fear (251, 252, 256), whereas dangers that cannot be controlled, may produce harm and elicit fear.

It is well established in human studies that fear comprises three loosely linked components: subjective feelings, fear-related behaviors including flexible changes and avoidance, and physiological arousal (248, 250, 257). The insula is well-suited to participate in the mediation of fear and its three components. For the subjective component, the insula represents bodily state (85, 258, 259), and is involved in cognitive processing (260, 261), so can generate bodily harm expectations. For behaviors, the insula projects via the corticospinal tract to spinal cord, and together with adjacent SII accounts for 3.4% of corticospinal projection neurons in monkey (262). Consequently, it can directly, rapidly, and efficiently modulate spinal motoneuron populations, so can generate flight and urgent motor behaviors. In addition, the insula is strongly functionally interconnected with ACC, including the cingulate motor area [CMA; Ref. (263–265)]. Further, a region of dorsal ACC (dACC) that likely includes part of CMA, was found to be reliably activated during fear experience in healthy humans, in a meta-analysis of fMRI neuroimaging studies (266). This brain area plays a major role in organizing diverse complex behaviors (195), including avoidance behaviors and emotional facial expression [see next section; Ref. (266, 267)]. Consequently, the insula can heighten appropriate behaviors, such effects being apparent during fear (268). For the physiological component, the insula sends substantial projections to ANS regulatory areas, and can modulate diverse autonomic functions (269).

Functional neuroimaging findings provide convergent support for insula mediation of fear (126, 243, 266, 270). Moreover, Caseras et al. (270) reported overlap of insula (and ACC) areas engaged in interoception and in fear. Another study compared fMRI activations across feared, pleasant, and unpleasant stimuli, and found enhanced insular subregion activation was specific for fear, whereas amygdala activation was common to multiple emotions (243). Further relevant observations are that the insula has moderate interconnections with PRC (120), so can contribute enduring fear representations to distributed representations. Despite the accumulated evidence, there is a scarcity of studies that directly investigate the contribution of the insula to fear, and such studies are needed. In sum, the insula commonly participates in the network that mediates PTSD, and it likely processes and represents the intense and persistent fear symptom of PTSD.

## Avoidance and Dorsal ACC

Dorsal ACC is activated in PTSD, according to findings from multiple neuroimaging studies [see for reviews, Ref. (16, 18)], and is suggested to mediate avoidance symptoms (Box 4f and related symptoms, Figure 2). The quantitative meta-analysis of Hayes et al. (16) covering all reviewed PTSD studies estimates the location of the activated dACC region, and it appears to encompass part of the CMA. The CMA (BA 24c) sends heavy projections to spinal cord motoneurons, M1, supplementary motor area, presupplementary motor area, premotor cortex, putamen, cerebellum, and lateral prefrontal cortex [lateral PFC; Ref. (264, 271)]. The CMA receives heavy to substantial inputs from diverse areas, including anterior cingulate gyrus (BAs 24a, 24b), insula, PRC, and lateral PFC (264, 271). The primate CMA comprises two subregions, both somatotopically organized, but with poorly understood functional differences, although the anterior subregion’s functions include representation of abstract aspects of behavior (271).

The ACC participates in networks that mediate wide-ranging motivated behaviors (195), including avoidance behaviors (272–274). In a rodent study, rats with freedom of movement were given mildly noxious stimulation when in part of a chamber, and neural activation was assessed by c-Fos expression. The findings were that the level of c-Fos expression in ACC was significantly and linearly related to behavioral avoidance of the noxious stimulation region (273). Rodent ACC is thought to correspond to primate BA 24 (275). In a study of surgical patients, painful laser stimulation was applied to the dorsum of the hand, and early neural responses were measured with chronically implanted intracerebral electrodes. The findings were that there were early activations within dACC, which was considered to correspond to CMA, and such activations were interpreted as mediating withdrawal/avoidance behaviors (272).

More generally, dACC is reliably activated in neuroimaging studies of pain, and has been suggested to mediate avoidance and other pain-related behaviors (274, 276). Also, dACC likely mediates avoidance during fear, as noted earlier. In addition, dACC activations during pain, fear, anger, other negative emotions, and cognition manifest substantial overlap (276). Thus, dACC and CMA representations likely support behavioral avoidance across multiple states. These representations may also support cognitive avoidance, in view of these regions’ involvement in cognitive processing (276), anterior CMA’s involvement in abstract aspects of behavior (271), and both regions’ heavy interconnections with lateral PFC (271). The CMA is also involved in emotional facial expression (267), which needs to be taken into account in theoretical and empirical studies. In sum, the commonly activated dACC region in PTSD which likely encompasses part of CMA, CMA’s heavy and extensive motor system projections, behavioral and neuroimaging findings across multiple states, and these regions’ cognitive functions and heavy interconnections with lateral PFC, together support dACC’s involvement in the behavioral avoidance and cognitive avoidance symptoms of PTSD. Moreover, the inputs to CMA arise from several regions involved in PTSD, suggesting such avoidance can be driven by several facets of PTSD.

## Predictions and Directions for Future Research

The proposed model of the hyperarousal subtype of PTSD, offers an account of how peritraumatic responses are transformed into an extensive array of the 20 DSM-5 PTSD symptoms, and is summarized in Figure 2. The amygdala is crucial to PTSD (79), and the model provides a causal account of amygdala activation, of insula activation, and explains how they influence diverse brain regions. The model thereby provides an etiological account of an array of PTSD symptoms, and explains the contributions to PTSD made by multiple brain regions, which are consistent with their known functions and connectivities, but whose contributions to PTSD were otherwise unresolved. The model is also consistent with findings that hyperarousal drives the other symptom clusters (33, 34), and that PTSD represents the extreme of a stress reactions continuum (42–44). Thus, the model integrates neuroscience and psychiatry to provide an extensive and dimensional account of a specific form of psychopathology, hyperarousal PTSD, and accords with the RDoC framework [cf. Ref. (40, 41)]. Together, the level of explanatory power and the number of symptoms elucidated exceeds those of existing accounts [cf. Ref. (17, 18, 21)], as summarized below. The model requires empirical tests in some areas, and also offers predictions and directions for future research, which should facilitate progress toward a comprehensive model of the hyperarousal subtype of PTSD, as also summarized below.

Traumatic events induce full PTSD in a proportion of those exposed, subthreshold PTSD in a larger proportion, and no disturbance in the majority; respectively, 12.5, 25.6, and 61.4% in a large Vietnam veterans sample (277), and this variation is an unresolved puzzle. The predominant existing models of PTSD involve fear conditioning supplemented by other concepts (18, 20–22), and thus focus on traumatic stimuli and their conditioning. In contrast, the current model proposes that peritraumatic responses are the major determinant of subsequent symptomatology, so the puzzle may be explained in part by variability of traumatic responses. That is, it is predicted that a given traumatic event may elicit differing levels of peritraumatic responses across individuals, leading to differing levels of subsequent symptomatology. A continuum of responses is well supported, and ranges from the adaptive to the extreme and maladaptive (42–44, 81). More generally, this hypothesis is consistent with the dimensional approach advocated by the RDoC framework (40, 41). An implication for prevention strategies is that amelioration of peritraumatic responses, in particular hyperarousal should ameliorate subsequent PTSD symptoms. There is some support for this from the preliminary findings that of a group of subjects (*n* = 11) intoxicated with alcohol at the time of trauma, none manifested early PTSD symptomatology (278). More generally, peritraumatic hyperarousal, stress hormones, heart rate, and so forth, need to be measured by real-time methods in empirical studies; current methods involve delays and other methodological shortcomings that likely produce significant underestimates, as noted earlier. Real-time measurements could be obtained remotely from personnel engaged in occupations with elevated rates of PTSD, and a prediction is that higher levels of peritraumatic arousal, stress hormones, and so forth, would be revealed than in existing studies. This seems likely as a significant proportion of combatants, for example, report such intense responses as faintness, vomiting, or defecation (148). Neuroanatomical, animal, and human neuroimaging studies suggest that multiple components of peritraumatic hyperarousal should heighten amygdala function, but empirical verification of this in human is needed.

Posttraumatic stress disorder develops in the absence of peritraumatic fear in a significant minority of patients [e.g., Ref. (23–25)], and this is difficult for existing models to explain. In contrast, the current model proposes that hyperarousal, which is processed by the multifunctional amygdala, is sufficient for the development of diverse PTSD symptoms (Figure 1), which are able to satisfy DSM-IV (279) diagnostic criteria for PTSD. That is, the model explains how PTSD developed in the absence of peritraumatic fear. Hyperarousal to trauma-related reminders likely arises because amygdala and brainstem projection sites participate in distributed trauma-related representations. This prediction could be assessed using neuroimaging optimized for measuring brainstem activity. A therapeutic implication is that functional lesions of the amygdala–brainstem pathway by deep brain stimulation should have therapeutic potential, and there is preliminary rodent evidence for this proposal (280). This may need to be combined with treatment to reduce hyperactivity of the insula [which also modulates arousal; Ref. (136, 137, 269)] in patients with involvement of severe fear or pain symptoms. A number of medications have shown some efficacy in ameliorating PTSD symptoms, but rational pharmacotherapy options require development (12, 281). The proposed model suggests some directions for progress toward this goal. Specifically, the amygdala is hypothesized to modulate multiple brain regions to produce a diversity of symptoms (Figure 1), and diverse neurotransmitters may be involved in these pathways [cf. Ref. (92)]. Once the neurotransmitters involved in such pathways and symptoms are determined, then corresponding antagonists are predicted to attenuate those symptoms. Likewise, amygdala hyperactivity may be driven by severe pain as well as hyperarousal, so may be activated by additional neurotransmitters that require additional pharmacotherapy. Thus, the model may open the possibility of efficient, personalized interventions, in which pharmacotherapy and other treatments are matched to a patient’s particular pattern of symptoms.

The amygdala tunes the selectivity of auditory cortex neurons in rodents. Corresponding tuning of visual (and other sensory) cortex neurons and consequent enlargement of visual trauma-related representations is predicted to occur in human PTSD patients. Such plasticity effects likely participate in visual symptoms of PTSD, and merit investigation. The processing and participation of physical pain and of mental pain/dysphoria in PTSD are elucidated by the current model, but are unexplained by most existing models. Mental pain may be the common feature of peritraumatic dysphoric emotions (e.g., fear, shame, guilt, sadness, and anger) that can contribute to PTSD development, and this deserves further investigation.

The numbing symptoms of diminished interest, withdrawal, and blunted emotions, are unexplained by most existing PTSD models. The function of rACC is interpreted as the regulation of the amygdala, but the mechanisms producing its dysfunction in PTSD are unexplained (17, 18, 21). In contrast, the current model predicts that amygdala hyperactivity drives rACC hypoactivation, and this can be investigated at several levels. At the neuroanatomical level, amygdala neuronal boutons that synapse with rACC neurons are predicted to be predominantly inhibitory, and so should mainly form symmetric rather than asymmetric synapses [cf. Ref. (170)]. Amygdala–rACC projections are denser and more extensive than the reciprocal ones (130, 168, 206). Quantitative estimates of these projections may provide an index of the relative frequency of recruitment, and thus of the predominant direction of activation. At the functional level, replications of Garcia et al. (207) and Pérez-Jaranay and Vives (208), would determine whether amygdala suppression of rACC is a robust finding. Such studies may perhaps be possible in humans with intracerebral electrodes implanted over these regions as part of presurgical investigations, combined with, for example, presentation of feared stimuli [cf. Ref. (272)]. Also, fear-elicited rACC hypoactivation should be abolished by amygdala lesions (e.g., through Urbach–Wiethe disease) or inactivation. The script driven imagery paradigm with PTSD patients may be combined with fMRI and effective connectivity analyses to further determine the direction of activation between rACC and amygdala. More generally, rACC hypoactivation is predicted to produce impaired motivation (and other numbing symptoms), and to be positively correlated with quantitative measures of impaired motivation, such as those of Robert et al. (282) and Starkstein et al. (283). More broadly, the above investigations could be extended to encompass mOFC, whose activation is also hypothesized to be suppressed by amygdala activity.

An unresolved puzzle is the explanation for the findings of Koenigs et al. (79), that lesions to vmPFC reduce the occurrence of PTSD. Lesions to ACC and OFC including vmPFC, and regions interconnected with them, produce apathy and disturbances of emotion and sensation [see earlier; Ref. (284)]. For example, failure to be offered food for 24 h, unexpected bereavement of a much loved spouse, or reminders of painful experiences, were found to produce little complaint or distress in single case studies of patients (196, 197). Substantial levels of pain assessed with the visual analog scale or other measures, are reported by some ACC lesioned patients not to be bothersome (285, 286). A testable speculation is that similar processes may operate in PTSD following lesions to vmPFC. That is, objectively measurable symptoms such as elevated physiological arousal and startle responses, may be regarded by the patients as not bothersome or distressing, so lowering the subjective ratings of these and other symptoms. Moreover, this pattern may be associated with some degree of apathy as assessed by apathy questionnaires.

Fear is poorly understood, and the widespread assumption of its activation simply by danger requires empirical testing. The insula appears to play a crucial role in the mediation of fear, and this may explain the findings that patients with amygdala lesions continue to experience fear (79, 246, 247). Accordingly, lesions or inactivation of the insula, for example, should cause impairment of fear, but currently there are few empirical studies of the insula’s contribution to fear. In addition, the insula and its projection sites can account for the three components of fear, but empirical tests are required. Avoidance behaviors are not explained by existing PTSD models (17, 18, 21), and the amygdala sends few projections directly to the motor system (111, 153). The current model, however, predicts that dACC activations derive from several facets of PTSD, that their locations encompass part of CMA, and that they likely mediate avoidance symptoms. This may be tested with more precise specification of the location of dACC activations in PTSD, whether this includes CMA, and whether this overlaps the location of dACC activations in pain, fear, and so forth. The dACC activations in PTSD may encompass one or both subregions of CMA, which could be localized and their functions broadly examined by means of motor and facial expression tasks in the same PTSD subjects.

Poor or negative social support after a traumatic event substantially promotes PTSD (20, 45, 46), and this effect requires explanation. Possible accounts are that poor or negative social support may bring about further stress and mental pain that exacerbate or reactivate existing PTSD symptoms [cf. Ref. (287)]. In contrast, positive social support may have corresponding ameliorative effects. There is limited literature on a number of PTSD symptoms (e.g., distressing dreams and distorted cognitions), so these require early investigation. More generally, enriched understanding of the mechanisms and symptomatology of hyperarousal PTSD should facilitate development of further novel prevention strategies and targeted therapies.

## Conclusion

A rich and valuable body of findings is accumulating on PTSD. The proposed theoretical model integrates wide-ranging findings about the hyperarousal subtype of PTSD into an extensive synthesis, which is detailed and parsimonious, and consistent with basic science and cognitive neuroscience literatures. It offers an account of the etiology and neurocircuitry of hyperarousal PTSD, explains a high proportion of the 20 DSM-5 PTSD symptoms, offers testable predictions to further illuminate the mechanisms of the disorder and of fear more generally, and has implications for prevention and treatment of the disorder.

## Conflict of Interest Statement

The author declares that the research was conducted in the absence of any commercial or financial relationships that could be construed as a potential conflict of interest.
